# Binding of Antitumor Ruthenium(III) Complexes to Plasma Proteins

**DOI:** 10.1155/MBD.2000.335

**Published:** 2000

**Authors:** L. Messori, F. Gonzales Vilchez, R. Vilaplana, F. Piccioli, E. Alessio, B. Keppler

**Affiliations:** 1 Department of Chemtstry University of Florence Via Capponi 7 Florence I-50121 Italy; 2 Department of Inorganic Chemistry University of Sevilla Aptdo. 553 Sevilla 41071 Spain; 3 Department of Chemical Sciences University of Trieste Via Giorgeri 1 Trieste 34127 Italy; 4 Institute of Inorganic Chemistry University of Vienna Wahringerstrasse 42 Wien A-1090 Austria

## Abstract

Presently, there is large interest in analysing the interactions *in vitro* with plasma proteins of some novel antitumor ruthenium(III) complexes that are in preclinical or clinical phase. The joint application of separation and spectroscopic techniques provides valuable information on the nature and the properties of the resulting ruthenium/protein adducts. Recent work carried out in our laboratory points out that, under physiological conditions, some selected ruthenium(III) complexes bind plasma proteins tightly with a marked preference for surface imidazole groups. Representative examples of interactions of antitumor ruthenium(III) complexes with plasma proteins such as albumin and transferrin are given. Notably the antitumor ruthenium(III) complexes considered here bind proteins much tighter than DNA; it is proposed that protein binding of ruthenium(III) complexes will have a large impact on the biodistribution, the pharmacokinetics and the mechanism of action of these experimental drugs.


### BINDING OF ANTITUMOR RUTHENIUM(III) COMPLEXES TO PLASMA PROTEINS

L. Messori*[1], F. Gonzales Vilchez[2], R. Vilaplana[2], F. Piccioli[1], E. Alessio[3] and B. Keppler*[4]

[1]Department of Chemistry, University of Florence, Via Capponi 7, I-50121 Florence, Italy
[2]Department of Inorganic Chemistry, University of Sevilla, Aptdo. 553, 41071 Sevilla, Spain
[3]Department of Chemical Sciences, University of Trieste, Via Giorgeri 1, 34127 Trieste, Italy
[4]Institute of Inorganic Chemistry, University of Vienna, Wahringerstrasse 42,A-1090 Wien, Austria

### ABSTRACT

Presently, there is large interest in analysing the interactions *in vitro* with plasma proteins of some novel antitumor ruthenium(III) complexes that are in preclinical or clinical phase. The joint application of separation and spectroscopic techniques provides valuable information on the nature and the properties of the resulting ruthenium/protein adducts. Recent work carried out in our laboratory points out that, under physiological conditions, some selected ruthenium(III) complexes bind plasma proteins tightly with a marked preference for surface imidazole groups. Representative examples of interactions of antitumor ruthenium(III) complexes with plasma proteins such as albumin and transferrin are given. Notably the antitumor ruthenium(III) complexes considered here bind proteins much tighter than DNA; it is proposed that protein binding of ruthenium(III) complexes will have a large impact on the biodistribution, the pharmacokinetics and the mechanism of action of these experimental drugs.

### INTRODUCTION

The importance of metal complexes in cancer chemotherapy is best documented by the results obtained with platinum(II) compounds; today cisplatin is one of the most used anticancer drugs against a wide range of malignancies including testicular carcinomas, ovarian tumors, head and neck cancers, bladder tumor and osteosarcoma [1]. Several detailed studies prove that the mechanism of action of platinum(II) complexes relies on the formation of covalent bonds with DNA [2].

Following the success of cisplatin, several metal complexes other than platinum have been considered over the years as possible alternatives to cisplatin [3]; in particular it was found that some ruthenium(III) compounds possess interesting antitumour and antimetastatic activities [4]. The field was pioneered by M.Clarke who synthesised and tested a variety of amminochloro ruthenium(III) complexes [4]. Later on, the group of Keppler prepared some new ruthenium(III) complexes, of general formula $RuCl_4L_2$, that turned out to be effective against a number of tumor models [5]. In the same years, the group of Mestroni in Trieste synthesized and characterized a large number of ruthenium(II) and ruthenium(III) mixed complexes with sulfoxide ligands [6]. Aminopolycarboxylate ruthenium(III) complexes designed on the basis of RuEDTA were prepared and tested in Sevilla by the group of Gonzalez Vilchez in the following years [7]. Additional ruthenium(III) complexes with promising biological properties were prepared in the laboratories of Peter Sadler [8] and Jan Reedjik [9]. As a result of such intense synthetic work, a large group ruthenium(III) complexes, of different structure, is now available that deserve extensive biological and pharmacological evaluation.

When studied *in vivo* some representative ruthenium(III) complexes were found to produce inhibition of DNA replication, mutagenic activity, induction of the SOS repair mechanism, binding to nuclear DNA and reduction of RNA synthesis [4]. To support a mode of action similiar to cisplatin, an "activation by reduction" mechanism was invoked according to which the inert ruthenium(III) complexes would act as pro-drugs that are activated *in situ* by reduction to the corresponding, more labile, ruthenium(II) species. The latter species is believed to be responsible of direct damage to DNA.

However, the results obtained so far point out that ruthenium(III) complexes bind DNA nucleobases much weaker than platinum(II) complexes; the conformational modifications of the double helix produced by ruthenium(III) complexes are generally modest [10].

The scarce binding ability of ruthenium(III) complexes for DNA together with the proved affinity of these complexes for proteins, suggests that the mechanism of action of ruthenium(III) complexes may be substantially different from that of platinum complexes; other crucial biomolecules, beyond DNA, might be the primary targets for ruthenium(III) compounds in cells.

Here we present experimental evidence, collected so far, documenting the favorable binding properties of selected antitumor ruthenium(III) complexes for plasma proteins such as albumin and transferrin. Among the several ruthenium(III) anticancer compounds prepared and tested up to now, we will specifically consider *trans*-Him[$RuCl_4(Im)_2$] (Im=Imidazole), **ICR** (figure 1/A) and *trans*-HInd[$RuCl_4(Ind)_2$],

**RuInd** (from the group of B. Keppler, Heidelberg) (figure 1/B) [11], Na[*trans*-$RuCl_4(Me_2SO)(Im)$], **NAMI** (from the group of Mestroni and Alessio, Trieste) (figure 1/C) [12], and dichloro-1,2-propylendiaminetetraacetate (PDTA)Ru(III), **RAP** (from the group of Gonzalez Vilchez, Sevilla) (figure 1/D) [13]. This choice is dictated by the fact that the selected complexes are among the most intensely studied ones and hold promise for future therapeutic applications. By the way we like reminding that NAMI A, the imidazolium salt of NAMI, is presently undergoing phase I clinical studies.

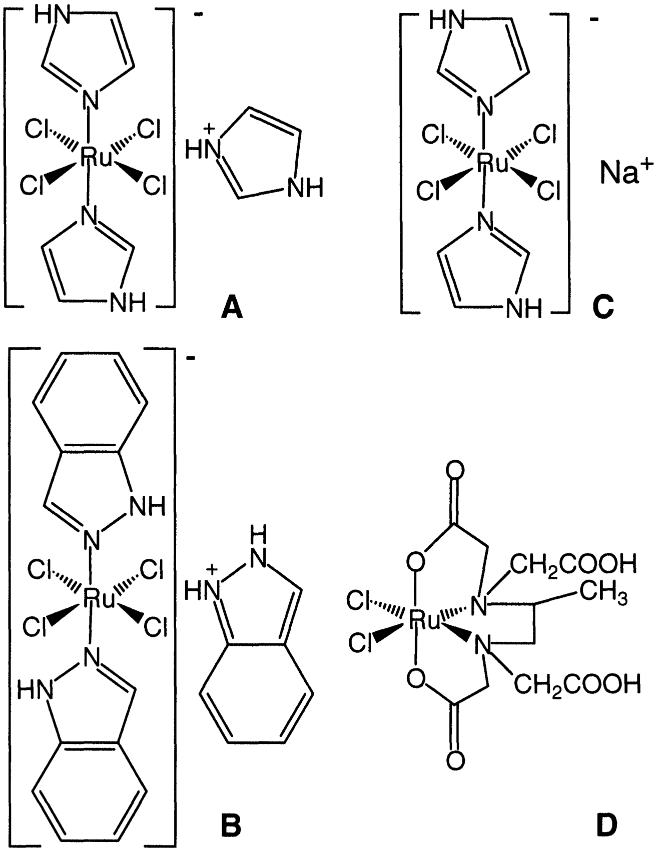

Figure 1: ICR (A), trans-Hind[RuCl4(Ind)2], NAMI (C) and RAP (D)

The interactions *in vitro* of these ruthenium(III) complexes with albumin and transferrin were investigated in detail by coupling classical separation techniques with spectroscopic methods such as spectrophotometry, circular dichroism and $^1H$ NMR. Indeed, the ruthenium(III) chromophore is particularly well suited for such sa pectroscopic approach since ruthenium(III) is paramagnetic and generally exhibits relatively intense charge transfer transitions in the visible. The studies carried out so far provide information on the nature and the strength of the ruthenium(III)/proteins interactions and on the possible biological consequences of such binding.

### BINDING OF RUTHENIUM(III) COMPLEXES TO PLASMA PROTEINS: SOME SELECTED EXAMPLES

*a) Binding of Keppler's compounds to the active site of human serum transferrin*

Keppler's complexes, ICR and RuInd, were the first ruthenium(III) complexes whose interactions with plasma proteins were considered in detail. Specifically, the interactions of both complexes with human serum transferrin were investigated in solution by various techniques, including $^1H$ NMR, HPLC, spectrophotometry and circular dichroism [11]. These studies have shown that either ruthenium(III) complex binds apotransferrin tightly, under physiological conditions, when working at 1:1 stoichiometry,. Ruthenium(III) binding occurs at the level of the iron binding site but heterocyclic ligands are conserved. Binding of ICR to apotransferrin is slow and takes place following hydrolysis of ruthenium coordinated chlorides; binding of RuInd is much faster and is facilitated by the presence of sodium bicarbonate in solution. Remarkably, the observation of a characteristic CD spectrum in the visible provides evidence for the formation of specific adducts between Keppler's ruthenium(III) complexes and apotransferrin. Spectra are shown in figure 2.

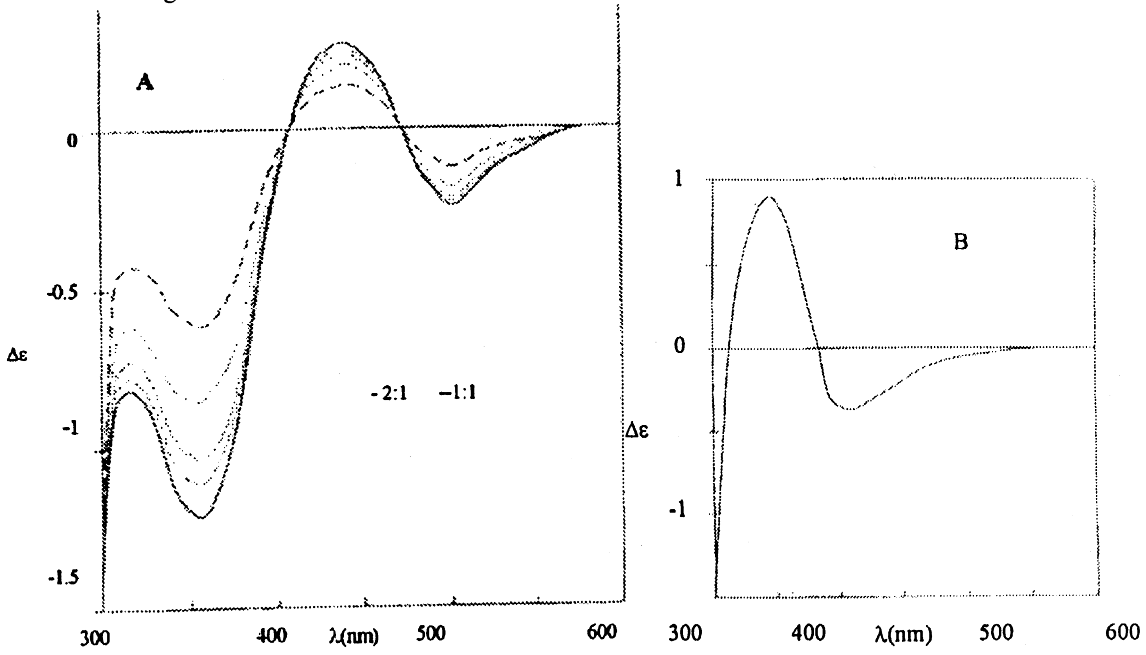

**Figure 2** CD spectra in the visible region showing the stepwise titration of apoTf (1x10-$^4$ M) with Rulnd (A), CD spectrum in the visible region of ru-im plus apoTf (1x10-$^4$ M) at a 2:1 ratio, obtained after 12h incubation.

Tight binding of these ruthenium(III) complexes to apotransferrin was demonstrated by HPLC methods; notably, it was observed that only under drastic conditions ruthenium(III) ions may be removed from apotransferrin (citric acid 0.1 M at pH 5.5) [11]. Later on, this description of the interaction of Keppler's complexes with apotransferrin was confirmed by X-ray diffraction studies carried out on adducts of apolactoferrin with Rulnd and ICR [14]. The latter studies showed that apolactoferrin can bind up to 5 ruthenium equivalents; binding takes place specifically at the iron binding sites and at exposed histidine residues (Figure 3). Structural details of the interaction of Rulnd with aminoacidic residues in the active site of apotransferrin are shown in figure 4.

*b) The reaction of NAMI with BSA*

We have recently described the reaction of NAMI A, with bovine serum albumin (BSA hereafter) monitored by various spectroscopic techniques [15]. We found that NAMI, following chloride hydrolysis, binds bovine serum albumin tightly; spectrophotometric and atomic absorption data pointed out that up to 5 ruthenium equivalents are firmly bound per albumin molecule when BSA is incubated for 24 hours with an eightfold excess of NAMI. Circular dichroism spectra showed that the various ruthenium centers bound to albumin exhibit well distinct spectroscopic features. The first ruthenium equivalent produces a characteristic positive CD band at 415 nm whereas the following NAMI equivalents give rise to less specific and less marked spectral effects. At high NAMI/BSA molar ratios a broad negative CD band develops at 590 nm. Notably the bound ruthenium centers remain in the oxidation state +3.

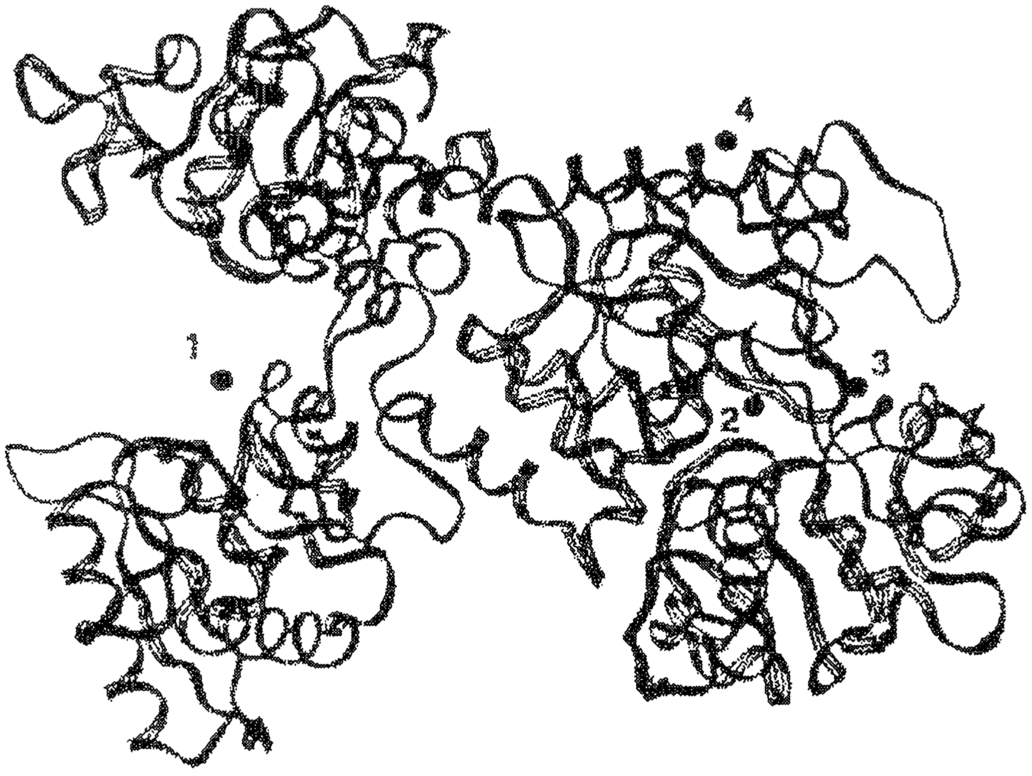

**Figure 3** Ribbon diagram showing of ru-im binding after soaking for 4 weeks. Sites are: (1) His 253, (2) His 597, (3) His 590, (4) His 654.

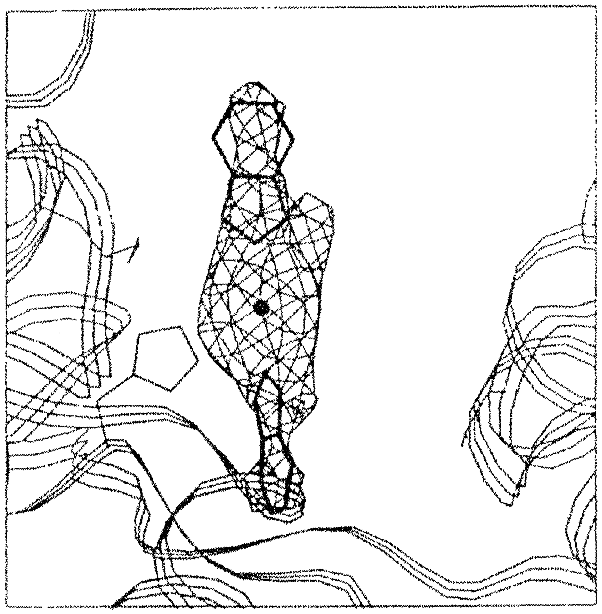

**Figure 4** Difference electron density for ru-ind in the N-terminal site of human apolactoferrin, showing that the two indazole ligands are retained. The Ru atom binds to His 253 and the nearby side chain of Lys 301 may help stabilize binding.

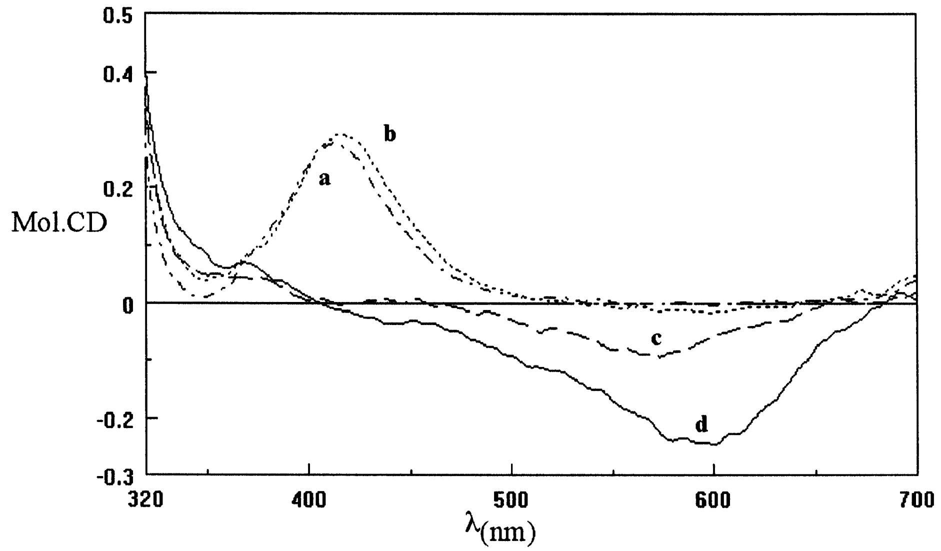

**Figure 5** CD spectra of NAMI/BSA samples. Samples were prepared at the following NAMI/BSA molar ratios: (a) 1:1, (b) 2:1, (c) 4:1 and (d) 8:1, and were measured after 24h incubation in buffer (phosphate 0.05M, NaCl 0.1M, pH 7.4, 25°C). Protein concentration: $1x10^{-3}$M.

By analogy with the case of transferrins it was proposed that the BSA-bound ruthenium ions are ligated to surface histidines of the protein; results from chemical modification experiments with diethylpyrocarbonate favor this view [15]. Spectral patterns similar but not identical to those shown by NAMI were observed when BSA was reacted with (sodium trans-bis(dimethylsulfoxide) tetrachloro ruthenate(III)) -Na[trans $RuCl_4$ $(DMSO)_2$] and with (ICR), implying a similar mechanism of interaction in all cases. Given the large abundance of albumin in the blood, it is likely that the described NAMI-BSA adducts may form *in vivo* and may be relevant for the biological properties of this complex and for its sequestration; alternatively NAMI/BSA adducts might act as specific carriers of the ruthenium complex to cancer cells. Similar results were found by Lemieska et al. when studying the interactions of ICR and RuInd with human serum albumin [16,17].

*c) RAP and PLASMA PROTEINS*

RAP is a promising polyaminocarboxylate ruthenium(III) complex with favorable antitumor properties. The solution behavior of RAP was characterised spectroscopically. Under physiological conditions RAP slowly looses the two coordinated chlorine atoms to produce a number of ruthenium (III) reactive species; the ruthenium ion always remains in the $3^+$ oxidation state and the PDTA ligand is always bound to the metal [18]. The reaction of RAP with bovine serum albumin, diferric transferrin or apotransferrin was investigated by spectrophotometry, circular dichroism and $^1$H NMR spectroscopy. For these studies, buffered solutions of RAP were reacted with bovine serum albumin, diferric transferrin and apotransferrin under a 1:1 ratio and the reaction mixtures analyzed through CD and $^1$H NMR spectroscopies. Surprisingly the CD spectra did not show any detectable band in the visible even after long incubation times in contrast to the cases of RuInd and *trans*-(DMSO) (Im)-tetrachlororuthenate. Instead, the $^1$H NMR spectra revealed significant changes of the hyperfine signals that occurred within a few minutes after mixing; noticeably, a the same variation in the pattern of the hyperfine signals was observed when reacting RAP with the three different samples. $^1$H NMR spectra of RAP with diferric transferrin (A), apotransferrin (B) and albumin (C) at 25°C are shown in figure 6

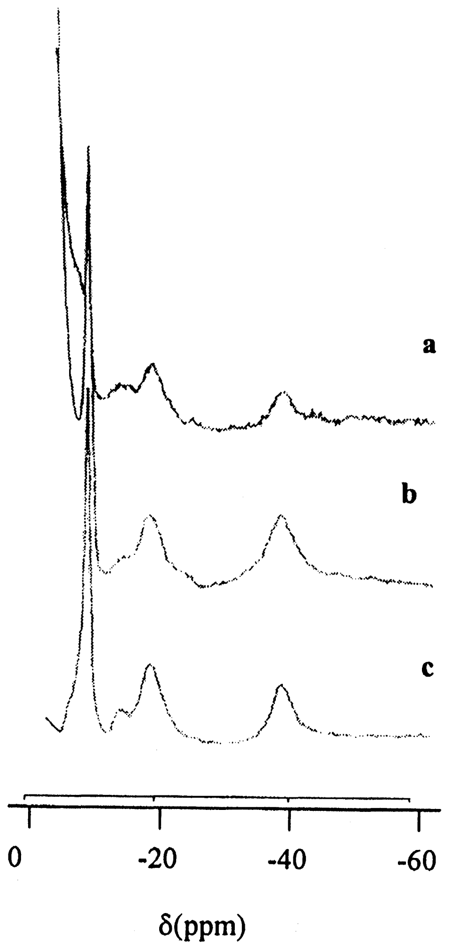

**Figure 6** Upfield region $^1H$ NMR spectra of RAP with diferric transferrin (a), apotransferrin (b) and albumin (c) at 25°C. Conditions: 50 nM phosphate buffer, pH 7.4; protein and RAP concentrations are in all cases 1 mM.

The occurrence of tight RAP-protein binding was then unambiguously demonstrated by a simple experiment: the solution containing diferric transferrin plus one equivalent of RAP was ultrafiltered through a Centricon with a cutoff of 10,000 daltons and the ultrafiltrate analyzed through $^1H$ NMR spectroscopy. No $^1H$ NMR hyperfine signals from RAP could be observed in the filtrate proving that most RAP (>90%) remains tightly bound to diferric transferrin. Noticeably, protein-bound ruthenium (III) could not be ultrafiltered even after addition of large amounts of sodium perchlorate (up to 0.5 M).

The virtual identity of the $^1H$ NMR spectra of the protein bound species in the three analyzed cases suggests a substantially equivalent mode of binding of RAP to the three proteins. It is apparent that, in all cases, the ruthenium ion remains in the $3^+$ state and that the PDTA ligand is still bound to the metal. Given the nearly equivalent mode of binding to the three proteins and the virtual absence of CD activity in the visible region, it was proposed that RAP binds to amino acid side chains that are exposed to the solvent on the protein surface. Again, a good candidate for binding is the imidazole ring of histidine which is known to represent a favorite binding site for ruthenium (III) ions.

### A GENERAL MODEL FOR THE REACTION OF ANTITUMOR RUTHENIUM(III) COMPLEXES WITH PROTEINS: PHARMACOLOGICAL IMPLICATIONS AND PERSPECTIVES.

The three examples reported above provide unambiguous evidence for the formation of stable adducts between selected antitumor ruthenium(III) complexes and plasma proteins. Studies carried out in our laboratory allowed us to identify some relevant common features of the resulting adducts that are listed below:

1. *Protein-bound ruthenium generally remains in the oxidation state +3.*
2. *binding is covalent and relatively tight -the ruthenium(III) chromophore may be displaced from the protein only under drastic conditions-.*
3. *binding most likely occurs at the level of histidine residues*
4. *kinetics of binding is relatively slow; binding occurs after displacement of ruthenium-bound chlorides.*

These observations emerging from spectroscopic results were nicely confirmed in the case of the adducts of Keppler's complexes with apolactoferrin by a single crystal X ray diffraction study. The latter investigation unambiguously proved that stable ruthenium/proteins adducts can form *in vitro*. Very recently Lobisky et al. have shown that RuInd binds human serum albumin faster and tighter than human serum transferrin; a large amount of the ruthenium complex can be removed from albumin by treatment with EDTA [19].

Undoubtely these results contain important pharmacological implications if one considers the growing role of ruthenium(III) complexes as possible antitumor drugs. Usually ruthenium(III) complexes in clinical trials are administered intravenously [20]. This implies that ruthenium(III) drugs will encounter very soon plasma proteins, in particular serum albumin that is very abundant in the blood stream. Given the relatively slow kinetics of protein binding, it is possible that a large amount of the ruthenium drug will escape albumin binding and will have the chance to spread within the organism in the "free" form; in any case a significant fraction of the injected drug will remain in the blood stream for enough time to bind plasma proteins, in particular albumin. So detailed studies are needed to establish whether protein-bound ruthenium is completely inactive or whether it may serve as a "reservoir" for the drug. A preliminary study of ours demonstrated that adducts of Keppler compounds with transferrin conserve some biological activity in vitro [21].

On the other hand the reported results provide evidence that ruthenium(III) complexes generally exhibit a good ability to bind proteins; indeed all the investigated ruthenium(III) drugs bind proteins much stronger than DNA [22]. This finding suggests that the important biological effects of antitumor ruthenium(III) complexes might be linked to direct damage of some specific and crucial protein rather than to DNA modifications. Further studies are needed to elucidate this critical issue.

**ACKNOWLEDGMENTS**
Cassa di Risparmio di Firenze is gratefully acknowledged for a generous grant.

**REFERENCES**

1. *Cisplatin*, Wiley-VCH, Weinheim, 1999;
2. Jamieson, E. R and Lippard, S. J. 1999, *Chem. Rev.*, 1999, **99**, 2467-2498;
3. *Metal Complexes in Cancer Chemotherapy* Keppler, B. K. (ed.) Weinheim: VCH, 1993;
4. Clarke, M. J., Zhu, F., Frasca, D. R., 1999, *Chem. Rev.*, 1999, **99**, 2511-2535;
5. Keppler, B. K., Lipponer, G., Stenzel, B., and Kratz, F., in *Metal Complexes in Cancer Chemotherapy*, Keppler B. K. (ed.) Weinheim: VCH, 1993, 187;
6. Alessio, E., Balducci, G., Lutman, A., Mestroni, G., Calligaris and Attia, W.M. *Inorg.Chim.Acta* 1993, **203**, 205;
7. Vilaplana, R.A., Gonzalez-Vilchez, F. and Ruiz-Valero, C. *Inorg. Chim. Acta* 1994, **224**, 15;
8. Guo, Z.J., Habtemariam, A., Sadler, P.J. and James B.R. *Inorg. Chim. Acta* 1998, **273**(1-2), 1-7;
9. Velders, A.H., Pazderski, L., Ugozzoli, F., Biagini Cingi, M., Manotti Lanfredi, A.M., Haasnoot, J.G. and Reedijk, J. *Inorg. Chim. Acta* 1998, **273**(1-2), 259-265;
10. Messori, L., Casini, A., Vullo, D., and Orioli, P., *Recent Research Developments in Inorganic Chemistry*, Transworld Research Network, in press.
11. Kratz, F., Hartmann, M., Keppler, B. and Messori, L. *J. Biol. Chem.* 1994, **269**(4), 2581-2588;
12. Sava, G., Alessio, E.,Bergamo, A. and Mestroni, G, 1999, in *Topics Biological Inorganic Chemistry*, Clarke, M. J., and Sadler, P. J. (eds.) Springer **1**, 143;
13. Vilaplana, R., Romero, M. A., Quiros, M., Salas, J. M. and Gonzalez-Vilchez, F., *Metal Based Drugs* 1995, **2**, 211;
14. Kratz, F., Keppler, B.K., Messori, L., Smith, C. and Baker, E.N. *Metal Based Drugs*, 1994, **1**, 169-173;
15. Messori, L., Orioli, P., Vullo, D., Alessio, E. and Iengo, E. *Eur. J. Biochem.* 2000, **267**, 1206-1213;
16. Trynda-Lemiesz, L., Karaczyn, A., Keppler, B:K: and Kozlowsky, H. *J Inorg Biochem* 2000, **78**(4), 341-6;
17. Trynda-Lemiesz, L., Keppler, B:K: and Kozlowsky, H *J Inorg Biochem* 1999, **73**(3), 123-8;
18. Vilchez, F.G., Vilaplana, R., Blasco, G. and Messori, L. *J. Inorg. Biochem.* 1998, **71**, 45-51;
19. Szpunar, J., Makarov, A., Pieper, T. Keppler, B.K. and Lobinski, R. *Anal. Chim. Acta* 1999, **387**, 135-144;
20. Kratz, F., Keppler, B.K., Hartmann, M., Messori, L. and Berger, M.R. *Metal Based Drugs* 1996, **3**, 15-23;

21 Kratz, F. in *Metal Complexes in Cancer Chemotherapy*, Keppler B. K. (ed.) Weinheim: VCH, 1993, 391-429;
22 Unpublished results from this laboratory.

**Received: January 11, 2001 - Accepted: January 21, 2001 -**
**Received in revised camera-ready format: January 22, 2001**

